# Alarm Burden in Infants With Bronchopulmonary Dysplasia Monitored With Pulse Oximetry at Home

**DOI:** 10.1001/jamanetworkopen.2022.18367

**Published:** 2022-06-23

**Authors:** Heidi M. Herrick, Molly Passarella, James Weimer, Christopher P. Bonafide, Sara B. DeMauro

**Affiliations:** 1 Department of Pediatrics, University of Pennsylvania Perelman School of Medicine, Philadelphia; 2 Division of Neonatology, Department of Pediatrics, The Children’s Hospital of Philadelphia, Philadelphia, Pennsylvania; 3 Roberts Center for Pediatric Research, The Children’s Hospital of Philadelphia, Philadelphia, Pennsylvania; 4 PRECISE Center, Department of Computer and Information Science, School of Engineering and Applied Sciences, University of Pennsylvania, Philadelphia; 5 Section of Hospital Medicine, The Children’s Hospital of Philadelphia, Philadelphia, Pennsylvania

## Abstract

This cohort study evaluates the association of settings on home oxygen saturation monitors with alarm incidence for infants with bronchopulmonary dysplasia (BPD).

## Introduction

Approximately half of extremely preterm infants have bronchopulmonary dysplasia (BPD).^1^ One-quarter of these infants are prescribed home oxygen,^2^ yet compliance with home pulse oximetry is low.^3^ Despite parental reports of frequent alarms contributing to premature monitor discontinuation,^4^ limited data exist to inform home alarm configuration and reduce unnecessary alarms. In a small observational study of a heterogenous pediatric population, longer alarm delays were associated with decreased alarm incidence.^5^ The true burden of home oximeter alarms and the impact of alarm parameters on alarm incidence are unknown in patients with BPD. Our objective was to evaluate the association of low oxygen saturation (Spo_2_) limits, alarm delays, and averaging times with alarm incidence by simulating threshold adjustments using data from a clinical trial of continuous home Spo_2_ monitoring among infants with BPD.

## Methods

The BPD Saturation Targeting Trial is an ongoing randomized clinical trial (NCT03385330) in which preterm infants with moderate or severe BPD undergo Spo_2_ monitoring (Masimo Rad-8) while asleep from 34 to 44 weeks’ postmenstrual age (PMA) to 6 months’ corrected age. In this secondary study, we used home oximeter data acquired during the first 4 weeks after discharge. Patients with 8 hours or more of usable data were included. Because no events requiring emergency intervention were reported while patients were on study monitors, all Spo_2_ readings of zero were considered artifact and excluded from analysis. We used the raw data to estimate counts of low Spo_2_ alarms that would occur with Spo_2_ limits of 80%, 85%, and 90%; alarm delays of 0, 5, 10, and 15 seconds; and averaging times of 8 and 16 seconds. We tested for linear trends in the rate of alarms per 8 hours (to estimate number of alarms per night) using the Cuzick test. Parents provided written informed consent for enrollment in this study approved by the Children’s Hospital of Philadelphia institutional review board. This study followed the STROBE reporting guideline.

## Results

Twenty infants with mean (SD) discharge PMA of 47.4 (5.2) weeks were included (Table). The Figure depicts the estimated number of alarms per 8 hours across Spo_2_ limits and alarm delays with 8- and 16-second averaging times. Under typical postdischarge oximeter settings at our institution (90% limit, 0-second delay, 8-second averaging time), patients would experience a median (IQR) of 23.1 (16.0-53.0) alarms per 8 hours. Patients would experience a median of less than 1 alarm per 8 hours with an Spo_2_ limit of 80%, 15-second delay, and averaging time of 8 or 16 seconds. Within each delay, lower Spo_2_ limits were associated with lower alarm rates for both 8- and 16-second averaging times (*P* < .001). Within a given Spo_2_ limit, longer alarm delays were associated with lower alarm rates (8-s averaging time: *P* < .001 for all Spo_2_ limits; 16-s averaging time: 80%, *P* < .001; 85%, *P* = .001; 90%, *P* = .003). With a 0-s delay and a 90% Spo_2_ limit, there was no significant difference in alarms per 8 hours between 8- and 16- second averaging times.

**Table.** Patient Characteristics

Characteristic	Patients, No. (%) (N = 20)
Birth gestational age, mean (SD), wk	26.1 (1.9)
Severity of BPD^a^	
Moderate	1 (5)
Severe	19 (95)
PMA at hospital discharge, mean (SD), wk	47.4 (5.2)
Respiratory support at hospital discharge	
Nasal cannula	10 (50)
Room air	10 (50)
Length of monitoring, mean (SD), h^b^	114.9 (80.7)

Abbreviations: BPD, bronchopulmonary dysplasia; PMA, postmenstrual age.

^a^ BPD severity defined according to 2001 Jobe and Bancalari National Institutes of Health Workshop. Moderate BPD was defined as 28 days of oxygen plus fraction of inspired oxygen of less than 30% at 36 weeks’ PMA or discharge; severe, 28 days of oxygen plus fraction of inspired oxygen of 30% or greater and/or positive pressure at 36 weeks’ PMA or discharge.

^b^ Length of nonzero oxygen saturation monitoring.

**Figure.**
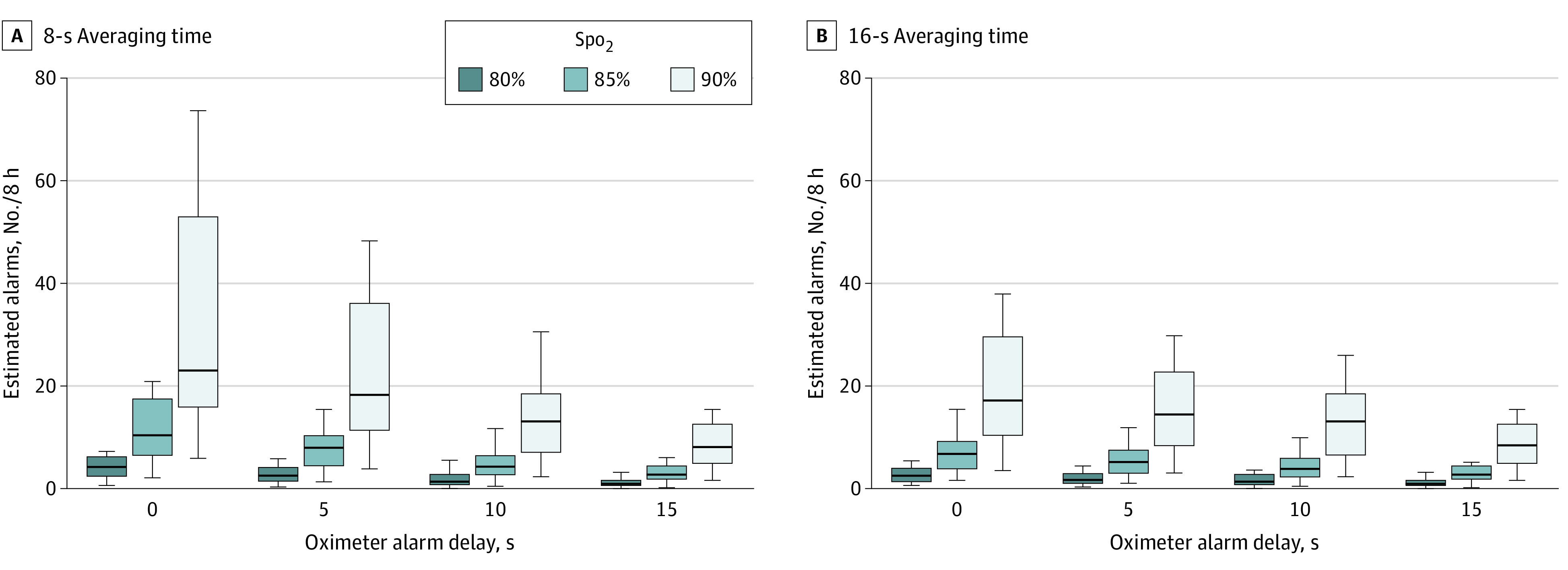
Low Oxygen Saturation (Spo_2_) Alarms Across Various Saturation Thresholds and Alarm Delays The boxes represent the IQRs, with the horizontal lines indicating the medians. Whiskers indicate the ranges.

## Discussion

We used patient-level data to demonstrate the burden of low Spo_2_ alarms among infants with BPD who are monitored at home. Furthermore, we showed the association of alarm parameters with alarm incidence. Current science suggests prolonged—but not necessarily brief—intermittent hypoxemia episodes with an Spo_2_ below 80% are associated with poor developmental outcomes in convalescing extremely preterm infants.^6^ Broadening Spo_2_ limits and/or adding alarm delays can significantly decrease alarm rates while preserving alarms for episodes of potentially critical hypoxemia. Typical home oximeter settings (90% limit, 0-s delay) seem to create an untenable alarm burden for caregivers. Study limitations include sample size and use of simulated alarms. Future studies need to assess the outcome of liberalizing home parameters to reduce alarm rates, which may include more uninterrupted sleep, decreased caregiver alarm fatigue, and improved compliance with home monitoring to optimize health and developmental outcomes.
